# Pressure point: blood flow restriction exercise and the pain paradox in musculoskeletal injury and persistent pain populations—a narrative review

**DOI:** 10.3389/fpain.2026.1822981

**Published:** 2026-06-16

**Authors:** Luke Gray, Luke Hughes, Lynn Kelly, Robert Barker-Davies, Russell Coppack, Nick Caplan, Robyn Cassidy, Sarah Lewis, Alexander Bennett, Peter Ladlow

**Affiliations:** 1 School of Sport, Exercise and Rehabilitation, Northumbria University, Newcastle, United Kingdom; 2 Academic Department of Military Rehabilitation, Defence Medical Rehabilitation Centre—Stanford Hall, Loughborough, United Kingdom; 3 Defence Medical Rehabilitation Centre—Stanford Hall, Loughborough, United Kingdom; 4 School of Sport, Exercise, and Health Sciences, Loughborough University, Loughborough, United Kingdom; 5 Department of Health, University of Bath, Bath, United Kingdom; 6 Faculty of Medicine, Imperial College London, National Heart and Lung Institute, London, United Kingdom

**Keywords:** blood flow restriction exercise, hyperalgaesia, hypoalgesia, musculoskeletal injury, pain modulation, persistent pain

## Abstract

Musculoskeletal injuries are commonly accompanied by acute and persistent pain; the latter of which can lead to maladaptive neurophysiological changes including central sensitisation and altered pain modulation responses. Blood flow restriction (BFR) exercise uses a pneumatic tourniquet to restrict arterial blood flow into the exercising limbs and has emerged as a promising rehabilitation tool, eliciting exercise-induced hypoalgesia (EIH) and increasing muscular strength and mass, but at lower external loads/intensities. The relationship between BFR tourniquet pressure and pain response is, however, complex. This review explores the mechanisms underpinning BFR-induced hypoalgesia and hyperalgesia across clinical and healthy populations. Findings indicate that while BFR exercise can reduce pain, potentially via metabolic, vascular, neurological, and psychological pathways, higher occlusive pressures or individual susceptibility, particularly in those with persistent pain, may provoke hyperalgesia. Mechanistically, this may involve inflammatory cytokine release, upregulated conditioned pain modulation and altered endorphin or endocannabinoid signalling. Psychological factors such as catastrophising and kinesiophobia may, furthermore, exacerbate nociceptive responses. These findings collectively highlight the pleiotropic and potentially hormetic nature of BFR exercise in pain modulation. Careful prescription, pressure selection, and patient monitoring are vital to maximise analgesic benefits while minimising adverse pain responses.

## Highlights

- Blood flow restriction (BFR) exercise can elicit exercise-induced hypoalgesia and comparable strength adaptations to traditional training, but at low external loads/intensities, offering value in musculoskeletal injury rehabilitation.
- Excessive occlusive pressures or altered pain modulation (e.g., central sensitisation, altered conditioned pain modulation) may shift the response from hypoalgesia to hyperalgesia, particularly in persistent pain populations.
- The effects of BFR exercise are pleiotropic and potentially hormetic in nature, requiring individualised prescription.

## 1 Introduction

Musculoskeletal injuries (MSKI) often present with an element of pain which, if present for more than 3 months, is deemed persistent pain (1, 2). With persistent pain comes a myriad of unfavourable neurophysiological maladaptation's including altered pain modulation [i.e., heightened central nervous system (CNS) sensitivity] (3) and centrally driven sensitisation [i.e., enhanced ascending nociceptive signalling within the spinal cord alongside impaired descending inhibitory control and/or increased descending facilitation from supraspinal centres (4, 5)], leading to altered pain processing (2, 6). Modern pain science recognises that biological injury and/or tissue damage cannot explain pain severity or persistence alone and embraces a biopsychosocial model to encompass psychological (e.g., cognition, mood and stress) and social factors (e.g., isolation or support) (7). In contrast to acute pain, persistent pain is now accepted as a disease state characterised by considerable neuroplastic changes, consistent with nociplastic pain (i.e., altered nociceptive processing within the central nervous system in the absence of clear ongoing tissue damage) and significant overlap with mood disorders (e.g., depression) (8–12). Additionally, there is a bidirectional relationship between persistent pain and psychosocial factors, whereby the neurophysiological maladaptation's of each, worsen the other (e.g., increased neuroinflammation) (13). These biopsychosocial factors do not operate independently but interact to influence both peripheral inflammatory responses and central pain processing (14). Psychological stress and mood disturbances have been linked to elevated pro-inflammatory cytokines (e.g., interleukin [IL]-6 and tumour necrosis factor-alpha [TNF-α]), while simultaneously altering central mechanisms, including reduced descending inhibitory capacity and enhanced facilitatory signalling (15). This integrated biopsychosocial–neuroimmune interaction contributes to heightened pain sensitivity and the persistence of pain, even in the absence of ongoing tissue damage (16, 17).

Given the interplay between biopsychosocial, inflammatory, and neurophysiological mechanisms, exercise is often used for rehabilitation following MSKI, and as a non-pharmacological method of alleviating acute and persistent pain (18–20). Wewege and Jones (21), however, reported no improvement in pain following exercise in individuals with persistent pain. Wider research suggests only modest effects on pain relief, and frequently zero difference between tissue-specific and non-specific exercises (22), and there is significant nuance to exercise modality [e.g., aerobic vs. resistance exercise (RE)] and clinical pathology (23). Exercise responses appear to vary according to pathology, symptom profile, and exercise prescription rather than anatomical location alone (24). Individuals with knee osteoarthritis, for example, have been shown to respond better to aerobic exercise (25), whereas people with chronic non-specific low back pain have shown more consistent improvements in disability and pain following resistance exercise (26). Individuals with fibromyalgia, however, have shown benefits with both aerobic and resistance exercise, the effects of which are maximised when combined (27). However, these findings should be interpreted cautiously, as the cited reviews included heterogeneous protocols with respect to exercise mode, intensity, supervision, and programme duration. Traditional exercise methods can be used to improve comorbidities commonly associated with MSKI and persistent pain (e.g., anxiety and depression) (19), but precautions must be taken with traditional exercise in MSKI rehabilitation due to a multitude of factors, such as arthrogenic muscle inhibition, kinesiophobia, and symptomatic impairment (28–31).

It is well documented that in individuals with persistent pain, there may be a reduced exercise-induced hypoalgesia (EIH) response or even elicit an hyperalgesic response following traditional exercise (32, 33); a phenomenon also seen within healthy populations (i.e., an individual free from acute, or persistent pain, clinical pathology, systemic or mental illness) (34). Exercise-induced hypoalgesia, mediated in part by the CNS, is defined as the temporary reduction in pain sensitivity during, or following, a single session of exercise and presents as a higher tolerance to a given exercise, task or stimuli, increased pain threshold, and/or lower reported subjective pain (33). Exercise-induced hyperalgesia is, conversely, an acute and transient increase in pain sensitivity following a single bout of exercise (19).

Blood flow restriction (BFR) exercise is an adjunct training method that partially restricts arterial inflow, and fully occludes venous outflow, in the working musculature by using a pneumatic torniquet system that applies an external pressure around the proximal region of the upper or lower limb (35). The amount of pressure required to completely occlude a limb's vasculature, defined as “limb occlusion pressure (LOP)”, is highly specific to the individual with factors such as limb size and tourniquet cuff shape, (i.e., straight or tapered) and compliance having influence” (36, 37). The application of pressure creates a hypoxic and metabolically stressful intramuscular environment that accelerates fatigue and increases metabolite accumulation, in turn amplifying the anabolic signalling and motor unit recruitment, amongst other physiological mechanisms, at low loads (38). Blood flow restriction exercise promotes similar morphological and physiological adaptations to those of resulting from traditional training methods, but at lower external loads and intensities (i.e., 20%–30% 1 repetition maximum [RM] or <50% maximal oxygen uptake [VO_2_ max]) (35, 39, 40). It has also been shown to elicit EIH during aerobic and resistance exercise (39, 41). Blood flow restriction exercise, therefore, creates an opportunity for load compromised populations (e.g., those with MSKI) to benefit from the low mechanical loads, promoting positive morphological and physiological adaptations within the rehabilitation process (42).

In healthy populations, BFR exercise has shown greater levels of EIH at the working site (i.e., quadriceps) in the exercising limb with low load RE (e.g., 30% 1RM) completed at higher relative pressures (e.g., 80% LOP) when compared to low- and high-intensity traditional RE methods (e.g., 30% and 70% 1RM) without BFR, as measured using pressure pain thresholds (41). High- and low-pressure BFR groups have both demonstrated comparable systemic effects to high-intensity resistance training (41). Similarly, during aerobic exercise, both low- and high-pressure BFR promoted greater EIH locally and systemically than low-intensity aerobic exercise (∼40% VO_2_ max) without BFR, with local effects exceeding, and systematic effects comparable to, those observed during high-intensity (∼70% V˙O_2max_ (39). Recent literature has demonstrated that BFR can be applied in isolation, as well as within submaximal, non-failure exercise paradigms, providing a clearer examination of the independent and dose-dependent effects of the occlusive stimulus (43–45). These findings suggest that the magnitude of hypoalgesia observed in earlier studies may reflect a combined effect of BFR and high-effort exercise, rather than the occlusive stimulus alone (46). This distinction is particularly relevant for clinical populations, where submaximal BFR prescriptions may offer a more tolerable and scalable approach, while still eliciting meaningful physiological and perceptual responses (43, 45). Collectively, this highlights that both occlusion pressure and exercise prescription (e.g., failure vs. submaximal loading) are key determinants of the resulting pain response (43, 46).

These findings have led to the suggestion that BFR exercise may not only promote increases in strength and function in load compromised populations, but also reduce pain symptoms (42, 47). Pain responses to BFR, however, appear to be complex and context-dependent depending on physiological, psychological, and methodological factors. This review discusses the evidence for BFR-induced hypoalgesia in clinical populations and explores a potential “pressure point” where hyperalgesia may be induced following BFR exercise.

## 2 BFR-induced hypoalgesia vs. hyperalgesia: evidence across populations

It is widely recognised that EIH may be attenuated in individuals with persistent pain compared to pain-free populations (32, 33). Such reductions, however, are typically relative to healthy controls and do not necessarily indicate an absence of a hypoalgesic response. Indeed, individuals with persistent pain may still experience meaningful reductions in pain following exercise, albeit of smaller magnitude (33). Importantly, EIH responses should ideally be interpreted relative to a non-exercise control condition; where such comparisons are not available, findings should be interpreted with caution (48). Furthermore, the assumption that greater hypoalgesia is inherently more beneficial may not be appropriate, particularly in clinical populations where tolerability and symptom response may be more relevant than maximal analgesic magnitude (32, 33).

Within clinical populations, there are numerous studies reporting a reduction of pain following BFR exercise. Resistance exercise with BFR (BFR-RE) has demonstrated context-dependent effects on pain; in a patellofemoral joint pain cohort, Giles et al. (49) reported that low-load BFR produced a significantly greater reduction in pain at 8 weeks during activities of daily living than traditional high-load RE, while Korakakis et al. (50) showed that a single bout of BFR-RE elicited a large immediate hypoalgesic effect across functional tasks (*d* = 0.88–1.32) that persisted for at least 45 min, an effect not reproduced by low-load RE alone. Studies involving post-operative anterior cruciate ligament reconstruction and knee osteoarthritis have, conversely found no additional pain-relieving benefit from BFR beyond traditional RE, despite improvements in strength and function (51, 52).

In chronic non-specific low back pain patients, Liu et al. (53) reported greater self-reported reductions in pain following 4 weeks of BFR-RE (70% LOP) vs. a non-occluded control group, indicating a global pain relieving effect. To date, however, there is a dearth of published research in clinical or healthy populations that show BFR exercise increases pain. Hyperalgesia may, therefore, be an issue that has not yet considered been by many. Li et al. (54) reported a dropout, due to excessive pain, following low-pressure BFR-RE (30% LOP) in older participants with knee osteoarthritis (*N* = 1/100). Additionally, Gray et al. (183) reported an increase in Brief Pain Inventory “worst pain” and “pain severity” pre-to-post intervention in the high-pressure BFR group (80% LOP, 7× sessions over 5 days) despite both groups having similar significant increases in pressure pain thresholds at local and systemic sites, showing distinct differences between acute objective changes and chronic subjective changes.

**Figure 1:**
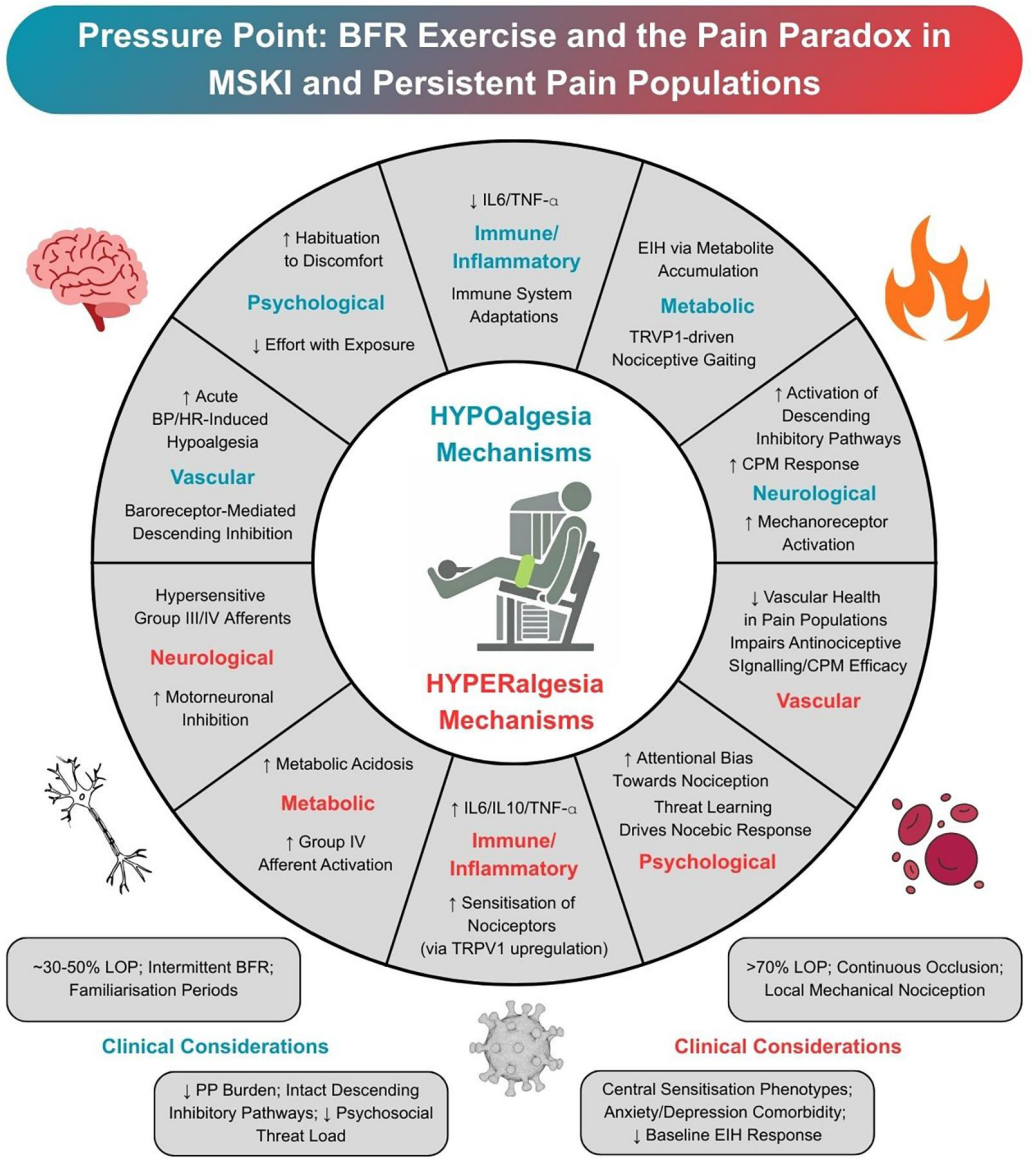
Schematic of mechanistic overview of BFR-induced hypoalgesia vs. hyperalgesia. BFR, Blood Flow Restriction; BP, Blood Pressure; CPM, Conditioned Pain Modulation; EIH, Exercise Induced Hypoalgesia; HR, Heart Rate; IL10, Interluekin-10; IL6, Interluekin-6; LOP, Limb Occlusion Pressure; PP, Persistent Pain; TNF-*α*, Tumour Necrosis Factor Alpha; TRVP1, Transient Receptor Potential Vanilloid 1. *Please note, “Motor Neuronal Inhibition” refers to an overarching collection of mechanisms discussed within the manuscript.

The variability in pain responses to BFR exercise, ranging from hypoalgesia to hyperalgesia, suggests that multiple interacting mechanisms are likely involved. Rather than being attributable to a single pathway, these responses likely emerge from the dynamic interplay between peripheral factors (e.g., metabolic stress and inflammation), central nervous system processes (e.g., ascending nociceptive signalling and descending modulation), and psychosocial influences (33, 55). Understanding these mechanisms is critical to explain why BFR may elicit analgesic effects in some contexts yet exacerbate pain in others. The following sections outline the key mechanistic domains that may underpin these divergent responses (Figure 1).

## 3 Mechanistic considerations for BFR-induced hypoalgesia and hyperalgesia

### 3.1 Immune system and inflammatory response

It is widely established that the immune and inflammatory systems have a complex inter-relationship with MSKI and persistent pain and, therefore, with hyperalgesia (56, 57). Following injury, pro-inflammatory cytokines (e.g., IL-6 and TNF-α) are released (58), leading to the activation of ion channels on primary sensory neurons (59). These ion channels, and specifically transient receptor potential vanilloid 1 (TRPV1), lead to the increased sensitivity of sensory neurons (60). Following tissue damage, damage-associated molecular patterns and initial cytokine additionally release recruit innate immune cells (e.g., neutrophils and macrophages) to the injured site (61, 62). These cells subsequently release additional pro-inflammatory cytokines to amplify and coordinate the repair response (63, 64). The associated inflammation is, in turn, a driving factor towards central and peripheral sensitisation, thereby increasing sensitivity to pain outputs, via neuroplastic changes within the spinal cord and supraspinal pathways (57, 65, 66).

The inflammatory response to BFR exercise appears to be highly context-dependent, varying according to exercise protocol, duration, and population. Acute BFR exercise has been shown to transiently increase pro- and anti-inflammatory cytokines (e.g., IL-6, IL-10, TNF-α), reflecting a short-term stress response to metabolic and ischaemic conditions (67, 68). In contrast, longer-term BFR interventions may promote reductions in systemic inflammatory markers (e.g., IL-6, TNF-α, CRP) and upregulation of endogenous antioxidant systems, suggesting an adaptive, anti-inflammatory effect over time (69–71). However, this response is not universal; in certain populations (e.g., older or metabolically compromised individuals), or when combined with higher training volumes or concurrent modalities, BFR may attenuate expected anti-inflammatory adaptations or even elevate inflammatory markers (72, 73). Collectively, these findings suggest that BFR exercise elicits a differential and potentially hormetic inflammatory response, whereby acute increases in inflammatory signalling may underpin longer-term physiological adaptation but may also contribute to transient nociceptive sensitisation depending on individual and methodological factors. Additionally, acute increases of norepinephrine were reported following BFR exercise when compared to non-occluded exercise (74, 75). Noradrenergic projections to the dorsal horn modulate nociceptive transmission, with norepinephrine acting predominantly via α2-adrenergic receptors to inhibit afferent input and reduce neuronal excitability (76, 77). This system forms a core component of endogenous pain modulation and has been implicated in exercise-induced hypoalgesia (78). In the context of BFR exercise, increases in circulating norepinephrine may therefore contribute to analgesic responses via activation of descending inhibitory pathways, although direct mechanistic evidence remains limited.

It could be theorised, therefore, that BFR exercise is pleiotropic in nature within MSKI and persistent pain populations. This notion, however, is also present with traditional exercise methods in those with persistent pain and MSKI; exercise can be both anti- and pro-inflammatory in nature, often related to both exercise intensity and volume (79, 80). Acutely, the metabolic (e.g., increased lactate and metabolic accumulation and activation of group III afferents) and mechanical (e.g., activation of group III afferents) stimuli associated with BFR may increase nociceptive input and contribute to transient local hyperalgesia (38, 81, 82). These responses are primarily mediated through metabolite-sensitive afferent pathways, with a potential, though less clearly established, contribution from local inflammatory processes (83, 84). Chronically, however, it may reduce the same inflammatory cytokines, thereby promoting homeostasis.

### 3.2 Metabolic

Heightened metabolic stress is hypothetically fundamental for adaptation to BFR exercise (38); Acute bouts of BFR exercise, however, induce robust metabolic alterations (e.g., significant increase in hydrogen ions, lactate and inorganic phosphate) (85). Research shows that BFR-RE elevates lactate levels above volume- and weight-matched non-occluded exercise, which can even exceed high-load RE when BFR-RE is completed at ∼30% 1RM and/or >60% LOP (86, 87). When BFR-RE is completed at ∼20% 1RM and/or at lower pressures (i.e., <50% LOP), however, the lactate increases are modest-to-indistinguishable from non-occluded intensity- and volume-matched exercise (87, 88). Differences in lactate accumulation, however, may reflect proximity to task failure and overall metabolic stress, rather than exercise intensity or volume *per se*. This distinction is particularly important given that many BFR protocols are performed to, or near, volitional failure, potentially confounding the interpretation of lactate as a primary mechanistic driver of pain responses (89).

Continuous BFR and, therefore, prolonged ischaemia and hypoxia, leads to greater metabolite accumulation, increasing the likelihood of nociceptor activation and sensitisation (90). Importantly, the predominant afferent pathways activated may differ between BFR and non-occluded exercise. In non-occluded conditions, moderate metabolic acidosis typically activates acid-sensing ion channels, contributing to transient muscle discomfort (91, 92). In contrast, BFR augments metabolite accumulation and local ischemia, increasing stimulation of metabolite-sensitive group III/IV afferents (38, 93). While more severe local metabolic stress may increase the likelihood of engaging TRPV1, particularly under conditions of pronounced acidosis (94, 95), the extent to which TRPV1 is preferentially activated during BFR in humans remains unclear. As such, BFR likely amplifies overall afferent input rather than selectively activating a single ion channel pathway (93, 95). Lactate also influences neuronal energy metabolism, responsible for the generation of action potentials and, during persistent pain states, there is an increased energy demand, leading to greater lactate production and utilisation, thereby further exacerbating pain (96). High levels of circulating lactate also enhance the activity of bradykinin (97), a potent pain generating substance and inflammation mediator (98). Additionally, lactate accumulation in the brain and spinal cord can lead to synaptic plasticity changes, upregulating glial cell expression and activating downstream signalling pathways, thereby maintaining persistent pain states (99).

### 3.3 Neurological

After the initial injury has occurred, a negative neurophysiological cascade occurs in response to the trauma (100). In some people, this can promote hyperalgesia and central sensitisation, typically arising from sustained or repeated nociceptive input leading to activity-dependent plasticity within dorsal horn neurons (65), resulting in increased sensitivity to stimuli at, and around, the injured site (100, 101). Through centrally mediated sensitisation and impaired descending pain modulation, the conditioned pain modulation (CPM) system may be impaired in persistent pain states, with a shift from effective descending inhibition towards enhanced descending facilitation, resulting in pain amplification, instead of inhibition (102). Conditioned pain modulation is an endogenous pain inhibitory mechanism commonly described as “pain inhibits pain”, whereby a noxious conditioning stimulus reduces the perception of a spatially remote test stimulus (103). This paradigm reflects the activation of descending inhibitory pathways originating from supraspinal centres, including the periaqueductal grey and rostral ventromedial medulla, which act to suppress nociceptive transmission at the spinal level (104). While CPM and EIH are often discussed in parallel, they differ in both experimental paradigm and underlying mechanisms (104). Although both involve activation of endogenous pain inhibitory systems, EIH likely represents a more complex, multi-system response involving metabolic, cardiovascular, and neurochemical factors in addition to descending modulation (19, 104). The CPM system has been reported as a potential mechanism for BFR-induced hypoalgesia (105, 106), however this has not been empirically and directly investigated to date. If the CPM system is upregulated in clinical pain populations, it is plausible that the noxious stimulus from BFR could promote hyperalgesia instead, especially when there is an association with increased sensitivity to mechanical stimuli (e.g., pressure) (4).

As individuals with persistent pain are often more sensitive to mechanical stimuli (107), BFR cuff width should be a key consideration for exercise prescription. Wider BFR cuffs (e.g., >10 cm) occlude at lower absolute pressure (108). Consequently, if not adjusted, this can inadvertently create a “high-mechanical pressure” condition and potentially provide a negative stimulus during rehabilitation. Consistency in cuff type and placement between LOP assessment and exercise is often advocated to improve accuracy of pressure application (109); however, direct empirical evidence supporting this recommendation remains limited. Given that cuff width, applied pressure, and exercise prescription interact to determine the overall stimulus, BFR should be considered a dose-dependent intervention (110), with these variables potentially contributing to variability in both hypoalgesic and hyperalgesic responses.

Higher BFR pressures (e.g., >70% LOP), additionally, restrict arterial inflow more substantially than lower pressures (e.g., 30%–50% LOP), causing greater ischemia, hypoxia and metabolite build up (111). Group III afferents (A-delta fibres) are thinly myelinated and primarily mechanosensitive, contributing to the perception of sharp pain or discomfort during muscle contraction; whereas, group IV (C fibres) afferents are unmyelinated, metabosensitive, and associated with the dull, burning and aching sensations linked to fatigue during and following exercise (93). Beyond their sensory function, these afferents play a critical role in regulating cardiorespiratory responses, facilitating increases in blood flow and oxygen delivery during exercise (112), while also contributing to central fatigue through reductions in motoneuronal output to limit excessive physiological strain (81). In persistent pain populations, these afferents may demonstrate increased sensitivity, potentially amplifying nociceptive input (11). During BFR exercise, the combined effects of mechanical compression and metabolite accumulation are likely to increase group III/IV afferent signalling (113). However, rather than acting as a direct driver of hyperalgesia, this heightened afferent input may serve as a peripheral trigger that interacts with central pain modulatory systems (114). As such, the resulting pain response may be context-dependent, reflecting the balance between descending inhibitory and facilitatory processes, and may manifest as either hypoalgesia or hyperalgesia. Additionally, there can also be a dysregulation of the endogenous endocannabinoid and opiate system of the body, in those with persistent pain (115), which, again, is a target pathway for BFR-induced hypoalgesia (105). Hughes and Patterson (41) and Hughes et al. (39) showed increases in beta-endorphin (an endogenous opioid) following BFR exercise in healthy populations; however, to our knowledge, these specific mechanisms have yet to be studied in a clinical population in relation to BFR and EIH.

### 3.4 Psychosocial factors

There is often a large psychological component with persistent pain (116). There is a strong bidirectional relationship between persistent pain and depression, evidenced with clinical overlap in presentation and shared anatomic regions of the brain to process elements of pain and depression (116). Anxiety and depression are both linked to increased levels of proinflammatory cytokines (e.g., CRP, IL-6, and TNF-*α*) (117, 118), further contributing to centrally driven sensitisation (119) which is, in turn, also associated with a heightened CNS excitability, characterised by increased ascending nociceptive signalling and/or reduced descending inhibitory control, relating to maladaptive changes to the serotonergic and noradrenergic systems (120). Noradrenergic projections from the locus coeruleus to the dorsal horn normally exert inhibitory effects via α2-adrenergic receptor activation, suppressing nociceptive transmission (77). A reduction in this inhibitory tone, or a relative increase in facilitatory α1-mediated signalling, may diminish endogenous pain inhibition (121). Similarly, serotonergic pathways originating from the brainstem (e.g., rostral ventromedial medulla) exert both inhibitory and facilitatory effects depending on receptor subtype and synaptic location; dysregulation of this balance may shift the system toward facilitation, further reducing pain modulation capacity (122, 123). Chronic psychological stress has also been shown to create a pro-inflammatory milieu, with cytokines IL-1B and IL-6 eliciting a hyperalgesic effect by activating nociceptors and central neurons (124). High levels of anxiety can, additionally, lead to a reduction in endogenous opioid function negating EIH response (118).

Catastrophising and kinesiophobia are key components of pain psychology, magnifying and reinforcing pain beliefs respectively (30, 125). Individuals who show greater traits of catastrophising pain often exhibit greater temporal summation of pain (i.e., a wind-up effect; a spinally mediated process within ascending nociceptive pathways characterised by progressive increases in dorsal horn neuron firing due to repeated C-fibre input, largely mediated by NMDA receptor activation) and diminished descending inhibition (126–128). Catastrophising induces a form of central hyperalgesia by keeping the brain focus on pain and preventing the activation of the endogenous pain relief pathways (129, 130). Sánchez-Sabater et al. (131) recently reported a moderate correlation between increased catastrophising and reduced EIH in older knee osteoarthritic patients. Previous research, however, has shown strong correlations between increased catastrophising and reduced EIH in both healthy populations (132, 133) and clinical populations (134). However, this relationship is not consistently observed, with some studies reporting no significant association between pain catastrophising and measures of pain modulation, including CPM and EIH (135, 136). This inconsistency suggests that the influence of catastrophising on pain modulation may be context-dependent, potentially moderated by factors such as population characteristics, pain status, and experimental paradigm. Chronic emotional stress can, additionally, activate the physiological stress response, leading to maladaptive changes that bias the brain to perceive experiences as threatening, potentially inducing hyperalgesia (137). While acute psychological stress can attenuate pain transiently, chronic or repeated psychological stress can lower pain thresholds and make minor aches feel more extensive (138).

Blood flow restriction exercise is known to be acutely uncomfortable because the tourniquet's constriction, and the associated reduced venous return, rapidly produce a tight, squeezing sensation alongside an early deep muscular burn or throbbing, and a sharp rise in perceived effort that is unpleasant, but typically tolerable and short-lived (35, 139–141). Research shows an increased stress and anxiety response following completion occluded vs. non-occluded low-load exercise (142); in turn, this may induce a nocebo effect, if inappropriately managed, leading to hyperalgesia (143). Anxiety and fear can heighten the attention to pain via physiological arousal (i.e., sympathetic activation, e.g., fight-or-flight), in turn exacerbating pain outputs (144).

Negative expectations can further worsen pain, as anxious anticipation triggers the release of neurochemicals such as cholecystokinin, which antagonise endogenous opioid-mediated analgesia and enhance pronociceptive signalling (145–147). This neurochemical shift may contribute to a transition from descending inhibitory to facilitatory pain modulation, thereby increasing pain sensitivity (148, 149). In this context, heightened anxiety may shift the balance of pain processing toward facilitation, increasing pain sensitivity. Increased psychosocial stress can tilt the balance from descending inhibition, to descending facilitation at the spinal and brainstem level (150). When activated by fear or stress, the amygdala can drive “on-cells” in the rostral ventromedial medulla that encourages dorsal horn neuron firing; in turn, generating greater pain from a given peripheral input (151). With sustained peripheral nociceptive input this may consolidate into central sensitisation via spinal plasticity (152). Whereas, in the absence of ongoing peripheral input it preferentially evolves into centrally driven sensitisation maintained by supraspinal network dysfunction (152–154).

Individuals with persistent pain have been shown to have an increased perceived effort, assessed through rate of perceived exertion, compared with healthy counterparts (155). This is pertinent, as BFR exercise has been shown to have a sharp rise in perceived effort (139–141). Over time, however, repeated BFR exposure has demonstrated lower perceived effort, with acclimatisation to the discomfort experienced during occluded exercise (156). Intermittent BFR (e.g., deflation during rest periods) has, additionally, been shown to lower perceived effort and pain when compared to continuous occlusion (157), especially when newly exposed to the BFR stimulus, and, therefore, may be appropriate for those with MSKI and/or persistent pain, with some research demonstrating similar positive morphological and physiological adaptations to continuous application (158). Additionally, cuff deflation during intermittent BFR may induce a reactive hyperaemic response, characterised by a transient increase in blood flow following reperfusion (159). This reperfusion phase may influence both local metabolic clearance and vascular signalling (160); therefore, may contribute to the variability in pain responses observed, as discussed in subsequent sections. The paradoxical relationship of inducing acute discomfort to relieve pain should, nevertheless, be a key consideration when prescribing BFR exercise in persistent pain populations, especially in those that exhibit signs of apprehension and kinesiophobia (161).

### 3.5 Vascular

Blood flow restriction exercise has been shown to increase blood pressure and heart rate during exercise (162), which has been associated with decreased sensitivity to noxious stimuli (163). Additionally, there is a potential overlap within the brain regions controlling baroreceptors and nociception, triggering descending inhibitory pathways to restore homeostasis (164). However, the extent to which these cardiovascular responses directly contribute to EIH during BFR remains unclear, and this mechanism has not been empirically demonstrated in this context. Acute increases in arterial blood pressure produces a heightened stretch of carotid and aortic baroreceptors, increasing afferent firing to the nucleus tractus solitarius (165). This baroreceptor-driven input engages clusters of nerve cell bodies within the brainstem (e.g., periaqueductal gray and rostroventral medulla) that control vital bodily functions (e.g., breathing and heart rate) (166, 167) to activate the descending inhibitory pathways that suppress nociceptive transmission (165, 168). Persistent pain is, however, often associated with cardiovascular dysfunction, including elevated blood pressure and heart rate at rest, as well as a reduction in heart rate variability (169). Research suggests that individuals with greater HRV demonstrate a more effective CPM response to noxious stimuli (170). Following MSKI, a pro-inflammatory environment is created in response to the damage and initiates the soft tissue healing cascade (171); however, if homeostasis does not occur, the pro-inflammatory environment can promote persistent pain and other negative physiological events (e.g., endothelial dysfunction) (57, 172).

Endothelial dysfunction has been reported in numerous MSKI and persistent pain pathologies, including chronic low back pain, fibromyalgia, osteoarthritis, and tendinopathy (173–176). Endothelial dysfunction reduces nitric oxide production and bioavailability, thereby creating a further pro-inflammatory environment (177) and an EIH response as nitric oxide has a central role in antinociception (178). Blood flow restriction exercise has a paradoxical relationship with endothelial function (179, 180). Acutely, BFR exercise abolishes the typical post-exercise increase in endothelial nitric oxide-mediated vasodilatation assessed via flow-mediated dilation, an effect attributed to increases in retrograde and oscillatory shear that suppress endothelial nitric oxide synthase signalling and reduce nitric oxide bioavailability (179, 180). While this attenuated flow-mediated dilation response could be viewed as unfavourable in younger, healthy adults with high endothelial responsiveness, its clinical relevance is less certain in older or rehabilitative populations who exhibit baseline endothelial dysfunction and reduced nitric oxide-mediated vasodilatory capacity (181, 182).

## 4 Conclusion

Blood flow restriction exercise offers a unique paradox within pain management and rehabilitation, with a potential hormesis effect. While it may promote hypoalgesia through metabolic, neurological, vascular, and psychological pathways, there is also potential for hyperalgesia, particularly in individuals with MSKI and/or persistent pain. Blood flow restriction exercise may induce hypoalgesia when pain is centrally sensitised but peripherally maintained, via engagement of descending inhibitory pathways, whereas in centrally driven sensitisation, where pain persists independent of peripheral input, the same afferent stimulus may fail to activate conditioned pain modulation and instead exacerbate pain through enhanced descending facilitation. The pleiotropic effects of BFR exercise, ranging from acute inflammatory and metabolic stress to chronic adaptive benefits, highlight the need for careful prescription and monitoring. Future work using n-of-1 trial designs may be particularly valuable in this context, as they allow for the systematic evaluation of individual-level responses to an intervention. Given the marked inter-individual variability in pain responses observed with BFR exercise, such approaches may help to identify responder and non-responder profiles and contribute to the development of more personalised and adaptive rehabilitation strategies for pain modulation.
